# Isolated Volar Dislocation of the Fifth Carpometacarpal Joint

**DOI:** 10.31486/toj.18.0151

**Published:** 2020

**Authors:** Bhumit Desai, Michael Nammour, Michael Warren, Gonzalo Sumarriva, Leslie Sisco-Wise

**Affiliations:** ^1^ The University of Queensland Faculty of Medicine, Ochsner Clinical School, New Orleans, LA; ^2^ Department of Orthopedic Surgery, Ochsner Clinic Foundation, New Orleans, LA

**Keywords:** *Carpometacarpal joints*, *closed reduction*, *isolated volar dislocation*, *joint dislocations*

## Abstract

**Background:** Isolated dislocation of the carpometacarpal (CMC) joints is a rare injury that accounts for less than 1% of hand injuries. Few cases of isolated volar dislocations of the fifth CMC joint have been reported, making such injuries worthy of reporting. Given the rarity of these injuries, they are easily overlooked in the emergency setting and thus require a high index of clinical suspicion.

**Case Report:** A 57-year-old female sustained an isolated volar dislocation of the fifth CMC joint when she fell onto her outstretched right hand. Physical examination revealed an inability to move the fifth digit, and the patient reported severe pain over the ulnar aspect of her right hand. X-rays of the right wrist revealed the dislocation. The patient was managed with closed reduction and application of an ulnar gutter splint.

**Conclusion:** Solitary dislocations of any CMC joint are less common than simultaneous dislocation of multiple CMC joints, especially at the fifth CMC joint with volar dislocation. Because of the potential long-term adverse effects of untreated dislocations, these injuries must not be overlooked. Thus, patients presenting to the emergency department after traumatic injury involving an axial loading force to the hand should be carefully evaluated.

## INTRODUCTION

Isolated dislocation of the carpometacarpal (CMC) joints is a rare injury that accounts for less than 1% of hand injuries.^1^ Joint dislocations in the hand typically require a bending component in the mechanism of injury, which can occur when the hand is trapped by an object and is unable to move with the rest of the arm. CMC dislocations are frequently seen in high-energy wrist trauma involving falls or motor vehicle injuries in which the joint was hyperextended.^2^ Few cases of isolated volar dislocations of the fifth CMC joint have been reported.^3-13^ Because of the rare nature of these injuries, they are easily overlooked in the emergency setting and thus require a high index of suspicion on the part of both clinicians and radiologists.

We report the case of an isolated volar dislocation of the fifth CMC joint and discuss the presentation and management.

## CASE REPORT

**Figure 1.**
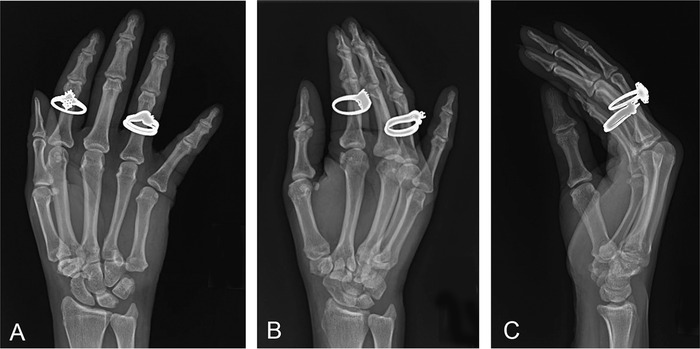
**Right hand posteroanterior (A), oblique (B), and lateral (C) x-rays at initial presentation in the emergency department. Fifth carpometacarpal joint dislocation was not identified.**

A 57-year-old female with sarcoidosis presented to the emergency department (ED) shortly after a fall from standing height. She reported bracing the fall with her outstretched right hand and complained of sudden-onset right elbow and right hand pain. She denied any loss of consciousness or any previous injuries to the hand or wrist. She reported an inability to move her fifth digit, with the worst pain over the ulnar aspect of the right hand. On physical examination, the patient was diffusely tender from the right elbow to the fingertips of the fourth and fifth digits. Swelling was minimal, with minor abrasions to the ulnar aspect of the right fourth digit. The right fifth digit was fixed in ulnar deviation and unable to perform full range of motion (ROM). The patient was noted to be neurologically and vascularly intact. The attending emergency physician ordered standard radiographs of the right hand (Figure 1), forearm, and elbow, but the radiologist did not initially identify the dislocation. The x-rays showed no other acute fractures or associated injury of the phalanges, metacarpals, wrist, or elbow.

**Figure 2.**
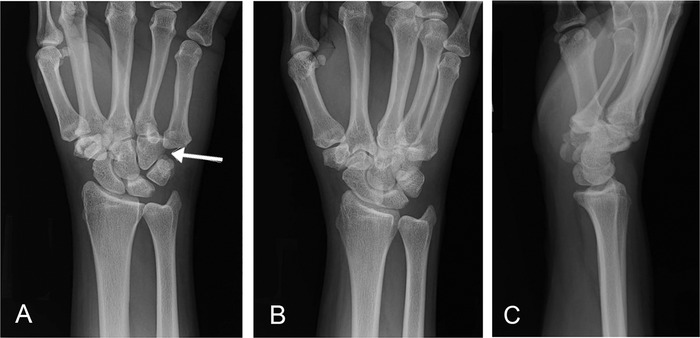
**Follow-up wrist posteroanterior (A), oblique (B), and lateral (C) x-rays in the emergency department confirmed an isolated volar and ulnar dislocation of the fifth metacarpal base.**

**Figure 3.**
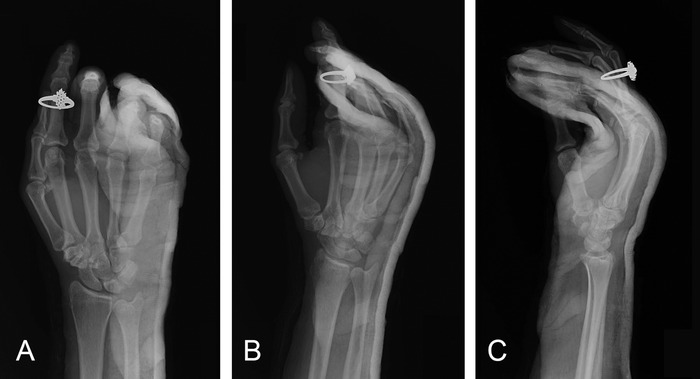
**Postreduction right hand posteroanterior (A), oblique (B), and lateral (C) x-rays show adequate alignment of the fifth carpometacarpal joint.**

The orthopedic team was consulted when the patient's ROM of the right fifth digit failed to improve. Subsequent wrist x-rays (Figure 2) confirmed an isolated volar and ulnar dislocation of the fifth metacarpal base. Closed reduction with local sedation was performed in the ED by applying longitudinal traction and dorsally directed force on the fifth metacarpal base. This technique resulted in satisfactory alignment that was confirmed by postreduction radiographs (Figure 3). The patient was placed in an ulnar gutter splint and discharged with follow-up scheduled in the orthopedic hand clinic.

## DISCUSSION

The first volar CMC dislocation was described in 1918.^3^ Since then, CMC dislocations have been well characterized in the literature. However, isolated volar fifth CMC dislocations are rarely recorded, making them worthy of reporting.^3-13^ Dislocations of the fifth CMC joint can be classified depending on dorsal or volar displacement.^14^ Dorsal dislocations, which primarily affect the fourth and fifth digits, are relatively more frequent than volar dislocations.^15^

Stability at the CMC joints is provided by a system of 4 ligaments, involving a high degree of anatomic variation among dorsal, multiple palmar, and 2 sets of interosseous ligaments.^16^ The index and long finger CMC joints are very rigid, allowing only 1 to 3 degrees of motion, while the CMC joints have looser ligamentous attachments that allow for greater mobility.^17^

Isolated dislocation of any CMC joint is far less common than simultaneous dislocation of multiple CMC joints. In the setting of multiple CMC joint dislocation, the fifth digit is involved in up to 80% of cases.^8^ Occult dislocations of the fifth CMC resulting from fourth metacarpal fractures or hamate fractures occur more commonly than isolated fifth CMC dislocations.^7^ In a descriptive study of 31 dorsal CMC dislocations of the fifth digit, only 1 case occurred without fracture.^18^ This study also found that 30% of the cases were missed initially but were identified retrospectively.^18^

Nalebuff proposed a classification system for isolated fifth CMC dislocations of the volar subtype according to the displacement of the fifth metacarpal base: radial palmar and ulnopalmar.^4^ The radial palmar dislocation is the more common subtype, and in this injury, all of the ligamentous and tendinous attachments are torn. In the rare ulnopalmar subtype, the pisometacarpal ligament and the flexor carpi ulnaris tendon attachments are intact. These 2 attachments pull the fifth metacarpal base proximally, volarly, and ulnarly.^8^ A thorough examination of the ulnar nerve function is imperative, as the nerve is susceptible to injury because of its proximity to the pisohamate ligament.^19^

When CMC dislocation is suspected based on clinical findings, posteroanterior (PA), oblique, and lateral x-rays of the hand should be obtained.^20^ The best approach to diagnosis uses a combination of all 3 views to arrive at the diagnosis. On PA plain films, dislocations should be suspected when there is disruption of Gilula arcs, loss of parallelism between CMC joints, or evidence of apparent shortening of the metacarpals.^20^ The PA view of the fifth metacarpal base should be over the hamate; ulnar offset of the fifth metacarpal in relation to the hamate suggests at least subluxation with possible dislocation. A fracture of a metacarpal base or a distal row carpal bone should increase the suspicion of a CMC dislocation.^8^ The lateral view must be appropriately taken, with congruent alignment of the radius-lunate-third metacarpal bone with the forearm in neutral position. A lateral view of the hand can characterize the dislocation as volar or dorsal, but dislocation can be concealed by overlapping of the joints. The PA view may be the only view in which an isolated fifth CMC dislocation can be seen and therefore requires significant attention to detail.^21^ If there is a high degree of clinical suspicion for CMC dislocation and x-rays are nondiagnostic, computed tomography scan is recommended.^22^

Surgical approaches to CMC dislocations include open reduction and internal fixation or closed reduction and percutaneous pinning. Nonsurgical methods such as closed reduction and conservative management with splint immobilization may be preferred if the patient has no concomitant lesions or has contraindications to surgery. Previous cases in the literature have reported excellent outcomes after 6 weeks of splint immobilization.^17^

Because of the potential long-term adverse effects of untreated dislocations, these injuries must not be overlooked. Underdiagnosis in the emergency setting may be attributed to other more obvious injuries taking precedence when patients present in a posttraumatic state. Because these injuries are uncommon and radiologic findings may be very subtle, they can be easily missed on initial evaluation as in the case of our patient. Untreated dislocations can result in chronic instability of the CMC joints and early articular degeneration.^23^ Other complications associated with CMC dislocation include imprecise alignment leading to chronic dislocation, posttraumatic arthrosis, median nerve dysfunction, carpal instability, complex regional pain syndrome, and tendon problems.

## CONCLUSION

Isolated pure dislocation of the fifth CMC joint, particularly the volar ulnar subtype, is a rare injury and highly susceptible to underdiagnoses in the emergency setting. Therefore, patients who present to the ED after traumatic injury involving an axial loading force to the hand should be carefully evaluated with a high index of suspicion. Management should involve immediate closed reduction in the acute setting. Definitive management can range from conservative to operative based on the severity of injury, adequacy of initial reduction, and involvement of surrounding structures.

## Notes

This article meets the Accreditation Council for Graduate Medical Education and the American Board of Medical Specialties Maintenance of Certification competencies for Patient Care and Medical Knowledge.
